# Anlotinib Combined with S-1 in the Third-Line Treatment of Stage IV Non-Small Cell Lung Cancer: Study Protocol for Phase II Clinical Trial

**DOI:** 10.31557/APJCP.2019.20.12.3849

**Published:** 2019

**Authors:** Xiyue Yang, Miao Xiang, Lidan Geng, Yixue Wen, Xiaobo Du

**Affiliations:** 1 *Department of Oncology, Mianyang Central Hospital, Mian Yang, *; 2 *Department of Oncology, Affiliated Hospital of North Sichuan Medical College, Nan Chong, China. *

**Keywords:** Anlotinib- S-1, Third-line treatment, Non-small cell lung cancer

## Abstract

**Background:**

A proportion of patients with stage IV non-small-cell lung cancer (NSCLC) is predicted to receive third-line treatment. However, currently no standard third-line treatment for NSCLC is available. Anlotinib is an oral, multi-targeted tyrosine kinase (TK) receptor inhibitor, which was approved as a third-line treatment for stage IV NSCLC in China on May 9, 2018. Nevertheless, The objective response rate of patients treated with anlotinib was merely 9.2% and the overall survival was only 3 months compared with the patients treated with placebo. Previous studies have shown that cancer treatment with a combination of chemotherapy with TK receptor inhibitors is effective and safe well tolerated. Therefore, the combination of anlotinib with other chemotherapeutic agents may be an effective treatment strategy for patients with stage IV NSCLC. Oral S-1 is a third-generation fluorouracil derivative; it showed good efficacy and caused relatively low toxicity in patients with NSCLC.

**Methods:**

The purpose of this trial is to evaluate the efficacy and safety of anlotinib combined with S-1 as the third-line treatment for patients with stage IV NSCLC. This is a prospective, phase II clinical trial. We will enroll29 patients with stage IV NSCLC treated with anlotinib plus S-1. Tumors will be assessed using computed tomography prior to treatment, after two, four, and six cycles of treatment, and during follow-up every 3 months until disease progression or death. The primary endpoint is the objective response rate (ORR). The secondary endpoints are progression-free survival, duration of response, proportion of disease control, and safety.

**Discussion:**

The expected outcome of this study is that anlotinib combined with S-1 has tolerable toxicity and better ORR than anlotinibmonotherapy. The results may indicate additional treatment options for patients with stage IV NSCLC.

## Introduction

Lung cancer has far-reaching medical, psychosocial, and economic impacts, and is a burden on the society (Rui et al., 2019).Worldwide, Lung cancer has the highest incidence and high mortality among all types of cancer; 2.1 million new lung cancer cases and 1.8 million deaths were predicted in 2018, which is approximately 1 in 5 (18.4%) cancer deaths (Bray et al., 2018). Non-small-cell lung cancer (NSCLC) accounts for ~85% of all lung cancer cases; majority of the patients are diagnosed after the disease has advanced or has become metastatic (Crinòet al., 2010; Felipet al., 2005; Williamet al., 2009).

The latest National Comprehensive Cancer Network (NCCN) Guidelines recommend gefitinib, lcotinib, erlotinib, and afatinib as the first-line treatment for stage IV NSCLC positive for epidermal growth factor receptor (EGFR) mutation. For patients with anaplasticlymphoma kinase (ALK) rearrangement or ROS proto-oncogene 1 (ROS1) fusion gene, crizotinib is recommended as the first-line treatment. First-line chemotherapy is recommended for advanced NSCLC without a driver gene; second-line treatment is used as a replacement of chemotherapy (National Comprehensive Cancer Network.,2019).Most patients with stage IV NSCLC are predicted to receive a third-line treatment; however, currently, there is no standard third-line treatment for NSCLC.

*K9*

Anlotinib is an oral, multi-targeted tyrosine kinase (TK) receptor inhibitor, which was approved as a third-line treatment for stage IV NSCLC on May 9, 2018 in China. However, the objective response rate (ORR) was only 9.2% and the overall survival (OS) time was prolonged only 3months (China Food and Drug Administration, 2018; Han et al., 2017; Han et al., 2018). The combination of multi-targeted TK receptor inhibitors and chemotherapy is effective and well-tolerated in patients with NSCLC and ovarian cancer (Nishioet al., 2013; Chun et al., 2018).Therefore, anlotinib combined with chemotherapy maybe an effective treatment strategy for patients with stage IV NSCLC. S-1 is an oral fluoropyrimidine agent containing the 5-fluorouracil, the prodrug tegafur, and two enzyme inhibitors, namely, 5-chloro-2,4-dihydroxypyridine and potassium oxonate; it showed good efficacy and caused relatively low toxicity in patients with stage IV NSCLC (Masuda et al., 2018; Iwamoto et al., 2015; Okumura et al., 2013).We designed this study to evaluate the efficacy and safety of anlotinib combined with S-1 as a third-line treatment for patients with stage IV NSCLC.

## Materials and Methods

*Study design*

The study design has been registered with the Chinese Clinical Trial Registry (Number: ChiCTR1900020948).This is a prospective, multicenter, phase II clinical trial. We will enroll 29 patients with cytologically or histologically confirmed stage IV NSCLC. The patients will be treated with anlotinib and S-1; they will undergo computed tomography (CT) before the treatment, after two, four, and six cycles, and during follow-up every 3 months until disease progression or death. If the efficacy is assessed as stable disease (SD), partial response (PR), and complete response (CR) after six cycles, anlotinib will be maintained until disease progression or death. Efficacy will be evaluated according to response evaluation criteria in solid tumors (RECIST); toxicity and side effects will be recorded. Statistical analysis of the data will be conducted using SPSS.

For this study, patients will be recruited from several hospitals in the Sichuan Province. There will be no randomized control group in this study. All patients who meet the inclusion criteria and provide informed consent will participate in the study.

*Objectives*

The primary endpoint is the ORR. The secondary endpoints are progression-free survival (PFS), duration of response, proportion of disease control, and safety.

*Inclusion criteria*

1. Aged 18–75 years

2. Pathologically diagnosed with late NSCLC

3. Eastern Cooperative Oncology Group (ECOG) performance scale (PS) score of0–1

4. Failure to respond to second-line treatment (patients with negative driver genes were evaluated for progression or intolerance after ≥ two different regimens of chemotherapy,and those with positive driver genes [EGFR mutation or ALK rearrangement] were evaluated for progression or intolerance after treatment with TK inhibitors and ≥ one regimen of chemotherapy)

5. A measurable lesion based on the RECIST criteria

6. The expected survival time≥12 weeks

7. Normally functioning major organs

8. Hemoglobin >100 g/L; platelet count > 100*10^9^/L; number of neutrophils >1.5 × 10^9^/L

9. Serum creatinine ≤1.25×upper limits of normal (ULN) or creatinine clearance rate > 60 mL/min.

10. Total bilirubin (TBIL) ≤1.5× ULN; as partate aminotransferase (AST)and alanine aminotransferase (ALT or SGPT) ≤2.5×ULN; alkaline phosphatase ≤ 5×ULN

11. Doppler ultrasound evaluation: left ventricular ejection fraction (LVEF)≥50%

12. No interstitial pneumonia or history of interstitial pneumonia

13. Patients or their family members who signed the informed consent

14. Female patients of childbearing age who agreed to use contraceptives (such as intrauterine devices, birth control pills, or condoms) in the study period and for six months after the end of the drug treatment; the serum or urine test indicated no pregnancy in the seven days prior to the study, and patients who were not lactating; male patients who agreed to use contraception during the study period and for six months after the end of the study period.

*Exclusion criteria*

1. Previously used anlotinib or S-1

2. Small-cell lung cancer (including small-cell carcinoma and non-small-cell carcinoma mixed lung cancer)

3. Presence of malignancies currently or in the past 5 years

4. Unable to intake medication orally (such as the inability to swallow, chronic diarrhea, intestinal obstruction.)

5. EGFR mutation-positive or ALK rearrangement-positive and no use the relevant targeted drugs

6. Brain metastases with symptoms or controlled symptoms for <two months

7. Central, empty lung squamous cell carcinoma or NSCLC with hemoptysis (>50 mL/day);

8. Severe and/or uncontrolled disease, including:

a. Uncontrolled high blood pressure (systolic blood pressure ≥ 150 mmHg and diastolic blood pressure ≥ 100 mmHg)

b. Myocardial ischemia or myocardial infarction and arrhythmia (QTc≥480 ms and ≥ 2 levels of congestive heart failure according to the New York Heart Association [NYHA]classification)

c. Active or uncontrollable serious infection (≥Common Terminology Criteria Adverse Events[CTCAE] level 2 infection)

d. Liver cirrhosis, decompensate liver disease, active hepatitis, or chronic hepatitis, which needs to be treated with antiretroviral therapy

e. Renal failure, which requires hemodialysis or peritoneal dialysis

f. History of immunodeficiency, including human immunodeficiency virus (HIV) or other acquired, congenital immunodeficiency disease, or history of organ transplantation

g. Poor control of diabetes (fasting blood glucose [FBG]> 10 mmol/L)

h. Routine urine test protein ≥++ and confirmed 24-hour urine protein> 1.0 g

i. Seizures that require treatment

9. Imaging studies shows that the tumor has invaded important vessels or the researchers determine that the tumor is likely to invade important blood vessels caused by fatal bleeding during the follow-up

10. Regardless of the severity, patients with any sign or medical history of bleeding four weeks prior to allocation, patients with any bleeding events ≥ CTCAE level 3, unhealed wounds, ulcers, or fractures

11. Arterial/venous thrombotic events(such as cerebrovascular accident [temporary ischemic attack], deep vein thrombosis, and pulmonary embolism) six months before allocation

12. Extra-field radiotherapy (EF-RT) performed four weeks before allocation, restricted radiotherapy performed for assessing tumor lesions two weeks before allocation or those prepared to receive local treatment (radiotherapy or radiofrequency ablation) at the time of the study; those undergoing palliative treatment, such as radiotherapy for bone metastases, will not be excluded.

13. Participated in other anti-tumor drug trials within four weeks

14. A history of psychotropic medicine abuse or mental disorders

15. Diagnosed with a disease that will severely endanger health or influence the completion of this study

16. Pregnant or lactating women

17. Considered unfit for inclusion by other physicians

*Data collection and management*

The following data will be collected from the patients: name, gender, age, lesion location, pathological type of cancer, clinical stage, drug dose and frequency, and examination results. We will review and analyze the data. We will acquire the data using an electronic data management system and generate a data base. We will perform statistical analysis before data audit.

*Sample size calculation*

We estimated the sample size using Simon’s phase II clinical trial design: with α = 0.05, power β = 0.2, and Pl– P0 = 0.20. The required number of patients was estimated to be ≥29based on the objective response rate of anlotinib monotherapy.

*Dose and frequency*

Anlotinib: orally 12mg once-a-day before breakfast for two consecutive weeks, then withdrawn for one week, with a 21-day course of treatment.

S-1: orally 70mg/m^2^/day, twice-a-day after meals for two consecutive weeks, then withdrawn for one week, with a 21-day course of treatment.

If the efficacy was assessed as SD, PR, and CR after six cycles; anlotinib was maintained until disease progression or death.

*Assessment of the primary and secondary endpoints*

Adverse events were recorded in accordance with the requirements of Medical Dictionary for Regulatory Activities (Med DRA). The severity of adverse events was graded according to the National Cancer Institute Common Terminology Criteria(NCI-CTC). The toxicity of the treatment, including acute and chronic toxicity, was evaluated during treatment and follow-up (Table 1).Any serious adverse drug reaction was promptly reported to the hospital ethics committee. PFS was defined as the date from randomization to tumor progression or death. Duration of response was assessed in patients who achieved a response and was defined as the time from the date of the first documented response to the date of the documented progression or death from any cause. Disease control was defined as the proportion of patients who achieved a CR, PR, or SD.

*Criteria for study discontinuation*

1. Withdrawal of informed consent

2. To ensure patients’ safety, the researchers may decide to discontinue the study

3. In case of an adverse event, researchers or patients may choose to discontinue the study

4. Researchers’ discretion

*Ethics*

The study was approved by the Ethics Committee of Mianyang Central Hospital, Sichuan, China (Number: S2019001). The study was supervised and managed by the ethics committee.

*Status*

We registered in the Chinese Clinical Trial Registry(Number: ChiCTR1900020948).Recruitment was initiated in January 2019, with a planned recruitment period of two years.

**Table 1:** Evaluation of the Treatment Process

Evaluation		Time
Hematological examination	Hemoglobin, platelets, white blood cells, neutrophils	Once a week
Biochemical tests	Biochemical and electrolyte	Once a week
Gastrointestinal effect	Nausea and vomiting	Once a week
Imaging examination	CT scan	Tumors were assessed before treatment, after 2 cycles, 4 cycles, 6 cycles, and during the follow-up every 3 months until disease progression or death.
	Brain MRI	Before treatment and every 3 months during the treatment and follow-up

## Discussion

There is currently no standard third-line treatment for advanced NSCLC. The vascular endothelial growth factor (VEGF) and its receptors (VEGFRs) are crucial in both vasculogenesis and angiogenesis, and have been proven as effective anticancer targets (Strumberget al., 2005; Mrosset al., 2012; Huang et al., 2012; Han et al., 2018). In a multicenter, randomized phase II trial, anlotinib was used as a third-line treatment; compared with placebo, anlotinib significantly improved PFS in patients with NSCLC, and the toxicity profiles showed good tolerance (Han et al., 2018). ALTER-0303 was a trial that compared the efficacy and safety of anlotinib with that of placebo in patients with advanced NSCLC who had progressed even after second-line treatment. 

S-1 is used for the treatment of NSCLC and to reduce the adverse effects of tegafur (Masuda et al., 2018). As S-1 is considered more effective than uracil-tegafur (UFT), long-term S-1 administration may be promising as an adjuvant chemotherapy regimen for advanced lung cancer (Iwamoto et al., 2015). Several studies have shown that the use of S-1 as adjuvant chemotherapy is associated with significant survival benefits in patients with NSCLC (Iwamoto et al., 2015; Okumura et al., 2013). The carboplatin and paclitaxel (CP) regimen is the standard chemotherapy regimen for advanced lung cancer (Schiller et al., 2002). In as tudy, the 5-year OS and disease-free survival were almost the same between the S-1 group and the CP group (Okuda et al., 2018). S-1 monotherapy also showed lesser hematotoxic adverse effects than other third-generation chemotherapy drugs. A randomized controlled trial in patients with NSCLC (East Asia S-1 Trial in Lung Cancer) confirmed S-1 as a viable treatment option for patients previously treated with NSCLC (Nokiharaet al., 2017). In ALTER-0303, each cycle of anlotinib treatment was defined as two weeks on-treatment followed by one week off-treatment; S-1 administration is the same with the anlotinib in combined regimen (Crinòet al., 2010; Han et al., 2018).

We believe that anlotinibin combination with chemotherapy would be an effective treatment strategy for patients with stage IV NSCLC. Therefore, we designed a prospective, phase II clinical trial to investigate the effectiveness and safety of anlotinibin combination with S-1; if this combination treatment is effective and well-tolerated, then it may be used as a third-line treatment for patients with stage IV NSCLC.

## Authors’ contributions

(I) Conceived and designed: X Du; (II) Administrative support: X Du; (III) Provision of study materials or patients: X Yang, M Xiang; (IV) Collection and assembly of data: X Yang, M Xiang, L Geng, Y Wen; (V) Data analysis and interpretation: X Yang, M Xiang; (VI) Manuscript writing: All authors; (VII) Final approval of manuscript: All authors.

## Data sharing statement

The informed consent documents are available on request.
